# The Henry Reaction in [Bmim][PF_6_]-based Microemulsions Promoted by Acylase

**DOI:** 10.3390/molecules181113910

**Published:** 2013-11-11

**Authors:** Wen-Jian Xia, Zong-Bo Xie, Guo-Fang Jiang, Zhang-Gao Le

**Affiliations:** School of Chemistry, Biology and Material Science, East China Institute of Technology, Nanchang 330013, China; E-Mails: xiawenjianhappy@126.com (W.-J.X.); gfjiang@ecit.edu.cn (G.-F.J.)

**Keywords:** Henry reaction, acylase, water-in-[Bmim][PF_6_] microemulsion, enzyme promiscuity

## Abstract

An environmentally-friendly, enzyme-promoted procedure for the Henry reaction was first studied using water-in-[Bmim][PF_6_] microemulsions as reaction medium. The Amano acylase from *Aspergillus oryzae* showed better catalytic activity for the addition reactions of nitromethane with a series of aromatic aldehydes, and a highest yield of 90% was obtained.

## 1. Introduction

The Henry (nitroaldol) reaction is considered one of the most powerful and atom economical C–C bond-formation reactions and is widely employed in organic chemistry. The resulting products, β-nitro alcohols, are often used as key intermediates in the synthesis of numerous biologically active compounds, including natural products, insecticides, fungicides and antibiotics [1,2,3,4,5]. Therefore, a variety of catalysts have been developed for this reaction, and basic catalysts are the most common ones [1,4,6,7,8]. However, this type of reaction is often complicated under strong alkaline conditions because of the unwanted side reactions, such as aldol, Michael and elimination reactions [6,9,10]. On the other hand, it's very gratifying to see that some Henry reactions have been performed under mild reaction conditions more recently, e.g., solvent free [11,12,13,14], in aqueous media [9,15,16,17] or using enzymes as catalysts [1,18,19,20].

Biocatalysis has become a powerful and useful tool for organic synthesis because of its high selectivity, mild reaction conditions and green features. Moreover, enzyme promiscuity has given a new push to its applications [21,22]. In 2006, the Griengl group reported the first example of a biocatalytic asymmetric Henry reaction catalyzed by hydroxynitrile lyase [18]. After that, aminoacylase [10], transglutaminase [19] and lipase [23] have also proved to exhibit nitroaldol activity. However, to the best of our knowledge, these enzyme-catalyzed Henry reactions were carried out in organic media, and the harm from organic solvents was unavoidable. Therefore, it is significant to develop new enzymatic methods for the Henry reaction in eco-friendly media. 

**Scheme 1:**
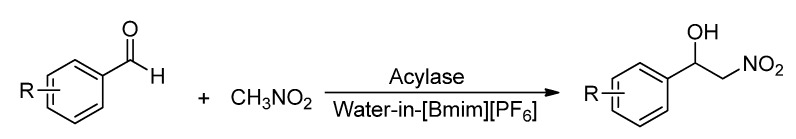
The acylase-catalyzed Henry reaction in water-in-[Bmim][PF_6_] microemulsions.

Thus, as one part of our continuing interest in enzyme promiscuity and green chemistry, we wish to report the first examples of biocatalytic Henry reactions in ionic liquid (IL)-based microemulsions (Scheme 1). ILs are usually considered as “green” solvents. Compared with ILs, water-in-IL (w/IL) microemulsions are more suitable for an enzyme-catalyzed conversion [24]. In microemulsions, the enzyme is located in the so-called “water pool” where it is exposed to a living environment similar to the natural one, thereby exhibiting good stability and activity [25,26,27]. At the same time, enzyme can be dispersed in the medium at a molecular level [24], which increases the odds of interaction between enzyme and substrate molecules. Here, 1-butyl-3-methylimidazolium hexafluorophosphate ([Bmim][PF_6_], a hydrophobic ionic liquid, was selected as oil phase, and the acylase-catalyzed Henry reaction in w/IL microemulsions was explored (Scheme 1). We were gratified to observe that excellent results were obtained in our preliminary study.

## 2. Results and Discussion

TX-100/H_2_O/[Bmim]PF_6_ microemulsions was first prepared according to the phase diagrams in literature [28]. Then the Henry reaction in microemulsions was studied selecting 4-nitrobenzaldehyde and nitromethane as a model reaction. Nine commercial enzymes were investigated to find the most suitable catalyst to catalyze the Henry reaction in w/IL microemulsions. As shown in Table 1, the best result of 88% yield was achieved by using Amano acylase from *Aspergillus oryzae* (AOA, entry 1), although other tested enzymes, even the non-enzyme protein bovine serum albumin (entry 10), also showed good catalytic activities. Next, some control experiments were performed to demonstrate the specific catalytic effect of the enzymes on the model reaction. The results showed that the model reaction could be performed smoothly in the microemulsions in absence of any enzyme (entry 11), although the yield was lower, but only a small amount of product was obtained under solvent-free conditions in the absence of enzyme (entry 12) and almost no product was produced in [Bmim][PF_6_] alone using AOA as a catalyst (entry 13). All these showed that this Henry reaction could be freely carried out in water-in-[Bmim][PF_6_] microemulsions, and AOA could effectively promote this reaction.

**Table 1:** Henry reaction catalyzed by different enzymes in IL-based microemulsions ^a^.

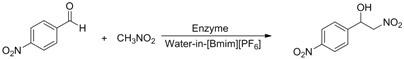		
Entry	Enzyme	Yield (%) ^b^
1	Amano acylase from *Aspergillus oryzae*	88
2	Lipase from *Rhizopus niveus*	72
3	Amano lipase PS from *Burkholderia cepacia*	71
4	Amano lipase from *Pseudomonas fluorescens*	70
5	Lipase from *Candida rugosa*	68
6	Lipase from bovine pancreas	68
7	Amano lipase M from *Mucor javanicus*	67
8	Amano lipase A from *Aspergillus niger*	66
9	Acylase I from *Aspergillus melleus*	65
10	Bovine serum albumin	64
11	Control test ^c^	62
12	Control test ^d^	24
13	Control test ^e^	1

^a^ *Reaction conditions*: Enzyme (30 mg), 4-nitrobenzaldehyde (1 mmol), nitromethane (4 mmol) and TX-100/H_2_O/[Bmim]PF_6_ microemulsions (1 mL, water 16.7%, TX-100 60%, [Bmim]PF_6_ 23.3%, in weight), was shaken at 30 °C for 48 h; ^b^ Determined by HPLC; ^c^ No enzyme; ^d^ Under solvent-free and enzyme-free conditions; ^e^ In 1 mL [Bmim]PF_6_ (with enzyme).

The influence of water content (ω_0_, where ω_0_ = [H_2_O]/[TX-100]) on the activity of AOA encapsulated in the “water pool” was investigated. W/IL microemulsions show a spherical droplet structure for which the droplet radius is directly proportional to the ω_0_ value and thus the microenvironment around the enzyme can be tuned by simply changing the ω_0_ value. Therefore ω_0_ is a key parameter and it plays a significant role in the enzyme-catalyzed reactions in w/IL microemulsions. It was found from the data listed in Table 2 that the ω_0_ value had an impact on this enzymatic reaction and the best result was obtained at ω_0_ = 10 (Table 2, entry 3).

**Table 2:** Effect of ω_0_ value on the Henry reaction ^a^.

Entry	ω_0_	Water (%)	TX-100 (%)	[Bmim]PF_6_ (%)	Yield ^b^ (%)
1	4	6.7	60	33.3	84
2	8	13.3	60	26.7	85
3	10	16.7	60	23.3	88
4	12	20.0	60	20.0	82
5	14	23.4	60	16.6	80
6	16	26.7	60	13.3	75

^a^ *Reaction conditions*: AOA (30 mg), 4-nitrobenzaldehyde (1 mmol), nitromethane (4 mmol) and TX-100/H_2_O/[Bmim]PF_6_ microemulsions (1 mL), was shaken at 30 °C for 48 h; ^b^ Determined by HPLC.

**Figure 1:**
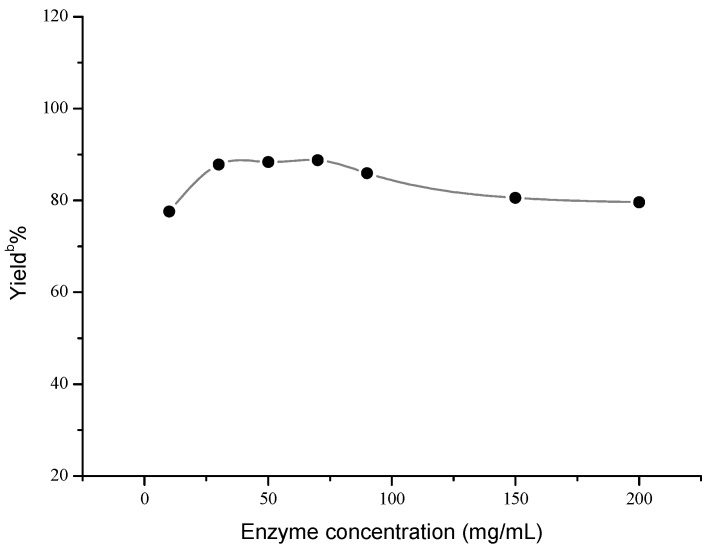
Influence of enzyme concentration on the Henry reaction ^a^.

^a^ *Reaction conditions*: AOA (10–200 mg), 4-nitrobenzaldehyde (1 mmol), nitromethane (4 mmol) and microemulsions (1 mL), was shaken at 30 °C for 48 h; ^b^ Yields were determined by HPLC.

**Figure 2:**
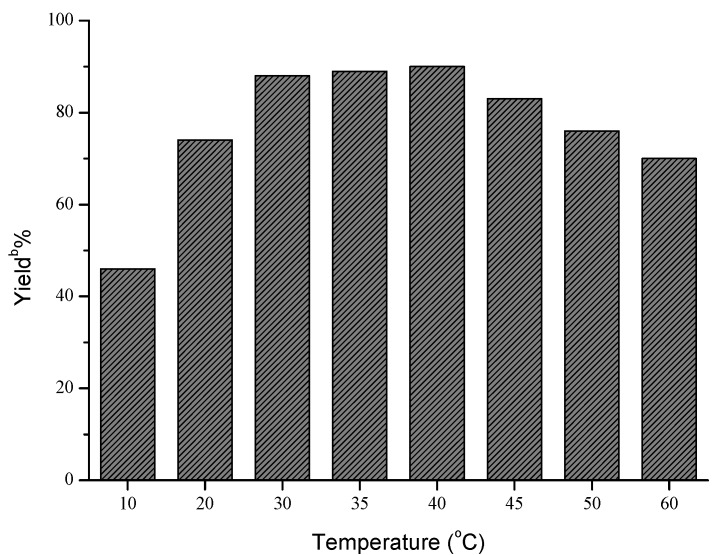
Influence of temperature on the Henry reaction ^a^.

^a^ *Reaction conditions*: AOA (30 mg), 4-nitrobenzaldehyde (1 mmol), nitromethane (4 mmol) and microemulsions (1 mL), was shaken at certain temperatures for 48 h; ^b^ Yields were determined by HPLC.

**Figure 3:**
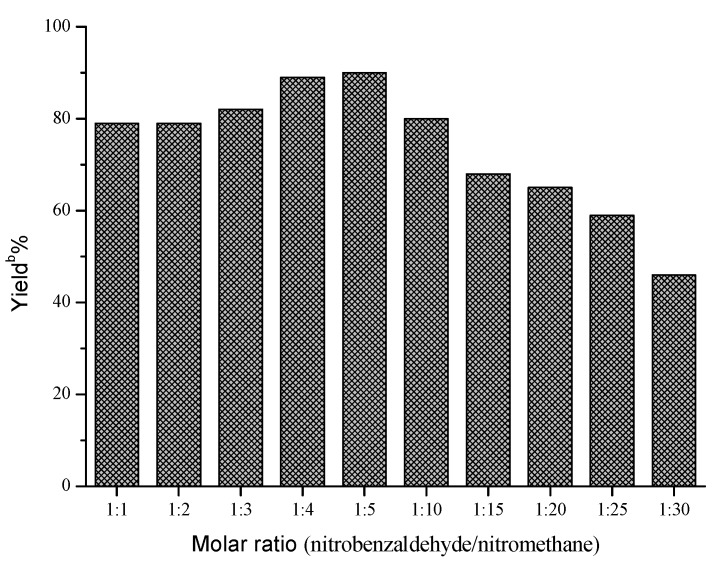
Influence of molar ratio on the Henry reaction ^a^.

^a^ *Reaction conditions*: AOA (30 mg), 4-nitrobenzaldehyde (1 mmol), nitromethane (1–30 mmol) and microemulsions (1 mL), was shaken at 40 °C for 48 h; ^b^ Yields were determined by HPLC.

Next, the influences of enzyme loading, temperature and molar ratio were studied respectively, and the optimal values of these factors were determined to be 30 mg/mL, 40 °C and 1:5, respectively. As shown in Figure 1, the effect of enzyme loading on the yield was not obvious, and the satisfying result could be obtained when 30 mg/mL AOA was adopted. However, temperature and molar ratio had significant effects on this reaction (Figure 2 and Figure 3). 

With the optimized conditions in hand, more aromatic aldehydes were evaluated to show the generality and scope of this new enzymatic promiscuity and the results are summarized in Table 3. It can be seen that a wide range of aromatic aldehydes can effectively react with nitromethane in water-in-[Bmim][PF_6_] microemulsions in the presence of AOA. In general, benzaldehyde and its derivatives bearing an electron-donating group gave the products in relatively lower yields (entries 1–3 and 7–10). In contrast, aromatic aldehydes containing an electron-withdrawing substituent provided β-nitro alcohols in higher yields. Especially, 2-nitrobenzaldehyde and 4-nitrobenzaldehyde (entries 4 and 6) gave the corresponding product in yields of 87% and 90%, respectively. This may be due to the fact that electron-withdrawing groups can enhance the electrophilicity of the carbonyl carbon in the aldehydes, which facilitates the reaction.

**Table 3:** Investigation of the reactant scope of the Henry reaction in IL-based microemulsions ^a^.

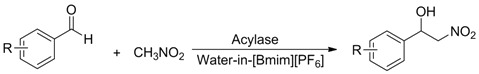			
Entry	R	Products	Yield ^b^(%)
1	H	*a*	44
2	3-CH_3_	*b*	28
3	4-CH_3_	*c*	30
4	2-NO_2_	*d*	87
5	3-NO_2_	*e*	79
6	4-NO_2_	*f*	90
7	2-OH	*g*	22
8	3-OH	*h*	48
9	4-OH	*i*	55
10	4-OCH_3_	*j*	40
11	2-Cl	*k*	77
12	4-Cl	*l*	66

^a^ *Reaction conditions*: AOA (30 mg), aldehydes (1 mmol), nitromethane (5 mmol) and microemulsions (1 mL), was shaken at 40 °C for 48 h; ^b^ Isolated yield after silica gel column chromatography.

## 3. Experimental

### 3.1. Materials and Analytical Methods

Amano acylase from *Aspergillus oryzae*, lipase from *Rhizopus niveus*, Amano lipase PS from *Burkholderia cepacia*, Amano lipase from *Pseudomonas fluorescens*, lipase from *Candida rugosa*, Amano lipase M from *Mucor javanicus*, Amano lipase A from *Aspergillus niger* and acylase I from *Aspergillus melleus* were purchased from Sigma-Aldrich Co. LLC (Shanghai, China). Lipase from bovine pancreas and bovine serum albumin were purchased from Aladdin Co., Ltd. (Shanghai, China). Other reagents were obtained from commercial suppliers and used without further purification unless otherwise noted. The reactions were monitored by thin-layer chromatography and visualized using UV light. The ^1^H-NMR spectra were recorded on a Bruker 400 MHz instrument using CDCl_3_ as solvent. Chemical shifts (*δ*) were expressed in ppm with tetramethylsilane (TMS) as internal standard, and coupling constants (*J*) were reported in Hz. High performance liquid chromatography (HPLC) was carried out on Waters instrument (Waters 2489, 1525) using a C18 column (250 mm × 46 mm). All products are known compounds and characterization data is only given below for a selection of products. Column chromatography was performed on silica gel using ethyl acetate-petroleum ether as mobile phase.

### 3.2. General Procedure for Henry reaction

Aromatic aldehyde (1 mmol), nitromethane (4 mmol), acylase (30 mg) and microemulsion (1 mL, water 16.7%, TX-100 60%, [Bmim]PF_6_ 23.3%) were added in a 10 mL test tube, then shaken at 260 rpm and 40 °C. After completion of the reaction, enzyme was filtered off to stop the reaction, then the filtrate was extracted with ethyl acetate and the resulting crude product was purified by column chromatography (petroleum ether-ethyl acetate) to give the pure product.

### 3.3. Physical and ^1^H-NMR Data of Some Representative Henry Products

*2-Nitro-1-phenylethanol* (***a***)


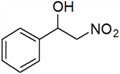


Yellow oil; ^1^H-NMR: δ = 7.39–7.33 (m, 5H), 5.40–5.36 (m, 1H), 4.58–4.51 (m, 2H), 2.86 (br, 1H). 

*2-Nitro-1-p-tolylethanol* (***c***)


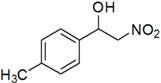


Yellow oil; ^1^H-NMR: δ = 7.27 (d, *J* = 7.94 Hz, 2H), 7.19 (d, *J* = 7.96 Hz, 2H), 5.44–5.38 (m, 2H), 4.62–4.48 (m, 1H), 3.65 (br, 1H), 2.37 (s, 3H).

*2-Nitro-1-(2-nitrophenyl)ethanol* (***d***)


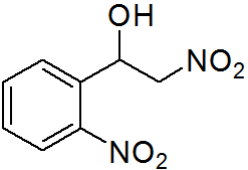


Yellow oil; ^1^H-NMR: δ = 8.07 (d, *J* = 8.0 Hz, 1H), 7.95 (d, *J* = 7.6 Hz, 1H), 7.75 (t, *J* = 7.4Hz, 1H), 7.55 (t, *J* = 7.6Hz, 1H), 6.04 (d, *J* = 8.8 Hz, 1H), 4.57–4.52 (m, 2H), 3.36 (br, 1H). 

*2-Nitro-1-(3-nitrophenyl)ethanol* (***e***)


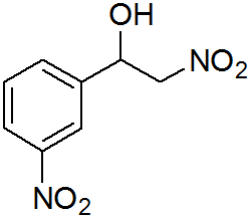


Pale yellow solid; ^1^H-NMR: δ = 8.26 (d, *J* = 8.8 Hz, 1H), 8.22 (d, *J* = 8.0 Hz, 1H), 7.77 (d, *J* = 7.6 Hz, 1H), 7.61 (d, *J* = 7.6, 8.0 Hz, 1H), 5.62–5.32 (m, 1H), 4.65–4.57 (m, 2H), 3.43 (br, 1H).

*2-Nitro-1-(4-nitrophenyl)ethanol* (***f***)


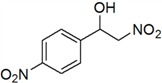


Yellow solid; ^1^H-NMR: δ = 8.26 (d, *J* = 8.8 Hz, 2H), 7.63 (d, *J* = 8.5 Hz, 2H), 5.24–4.76 (m, 1H), 4.52–4.22 (m, 2H), 3.65 (br, 1H).

*2-(1-Hydroxy-2-nitroethyl)phenol* (***g***)


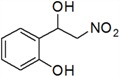


Colorless oil; ^1^H-NMR: δ = 7.25–6.98 (m, 4H), 5.66 (s, 1H), 4.59–4.51(m, 2H), 4.41–4.36 (m, 1H), 3.25 (br, 1H).

*4-(1-Hydroxy-2-nitroethyl)phenol* (***i***)


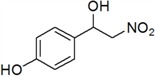


Yellow oil; ^1^H-NMR: δ = 7.56 (d, *J* = 9.4 Hz, 2H), 7.16 (d, *J* = 9.4 Hz, 2H), 5.31 (s, 1H), 4.68–4.61 (m, 2H), 4.58–4.34 (m, 1H), 3.82 (br, 1H). 

*1-(3-Chlorophenyl)-2-nitroethanol* (***k***)


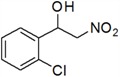


Pale yellow oil; ^1^H-NMR: δ = 7.66 (d, *J* = 7.6 Hz, 1H), 7.39–7.26 (m, 3H), 5.85–5.81 (m, 1H), 4.68–4.65 (m, 2H), 3.18 (br, 1H).

*1-(4-Chlorophenyl)-2-nitroethanol* (***l***)


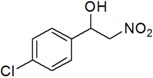


Yellow oil; ^1^H-NMR: δ = 7.38–7.27 (m, 4H), 5.43–5.39 (m, 1H), 4.57–4.54 (m, 2H), 3.11 (br, 1H).

## 4. Conclusions

In summary, an efficient and environmentally-friendly method has been developed for the Henry reaction, which is another example of biocatalytic promiscuity. Here, water/[Bmim]PF6 reverse microemulsion was used for the Henry reaction for the first time and AOA could effectively promote this reaction. As a green approach, it not only expands the applications of IL microemulsions, but might push forward the development of other reactions taking advantage of enzymatic promiscuity.

## References

[1] Milner S.E., Moody T.S., Maguire A.R. (2012). Biocatalytic Approaches to the Henry (Nitroaldol) Reaction. Eur. J. Org. Chem..

[2] Kisanga P.B., Verkade J.G. (1999). P(RNCH_2_CH_2_)_3_N: An efficient promoter for the nitroaldol (Henry) reaction. J. Org. Chem..

[3] Heffner R.J., Jiang J., Joullie M.M. (1992). Total synthesis of (−)-nummularine F. J. Am. Chem. Soc..

[4] Saraswat A., Sharma L., Singh S., Siddiqui I., Singh R. (2013). Chemoselective Henry reaction catalyzed by electro-generated base. Res. Chem. Intermed..

[5] Jiang T., Gao H., Han B., Zhao G., Chang Y., Wu W., Gao L., Yang G. (2004). Ionic liquid catalyzed Henry reactions. Tetrahedron Lett..

[6] Rokhum L., Bez G. (2012). Ethyl acrylate conjugated polystyryl-diphenylphosphine—An extremely efficient catalyst for Henry reaction under solvent-free conditions (SolFC). Can. J. Chem..

[7] Khan F.A., Dash J., Satapathy R., Upadhyay S.K. (2004). Hydrotalcite catalysis in ionic liquid medium: A recyclable reaction system for heterogeneous Knoevenagel and nitroaldol condensation. Tetrahedron Lett..

[8] Simoni D., Rondanin R., Morini M., Baruchello R., Invidiata F.P. (2000). 1,5,7-Triazabicyclo[4.4.0]dec-1-ene (TBD), 7-methyl-TBD (MTBD) and the polymer-supported TBD (P-TBD): Three efficient catalysts for the nitroaldol (Henry) reaction and for the addition of dialkyl phosphites to unsaturated systems. Tetrahedron Lett..

[9] Bora P.P., Bez G. (2013). Henry reaction in aqueous media at neutral pH. Eur. J. Org. Chem..

[10] Wang J.-L., Li X., Xie H.-Y., Liu B.-K., Lin X.-F. (2010). Hydrolase-catalyzed fast Henry reaction of nitroalkanes and aldehydes in organic media. J. Biotechnol..

[11] Majhi A., Kadam S.T., Kim S.S. (2009). TMEDA catalyzed Henry (nitroaldol) reaction under metal and solvent-free conditions. Bull. Korean Chem. Soc..

[12] Ballini R., Bosica G., Parrini M. (1998). A one pot, solvent-free synthesis of acyclic α-nitro ketones through the nitroaldol reaction. Tetrahedron Lett..

[13] Changsheng R.M.-A.H.R., Gan X.C., Lai G., Wang Z. (2006). Rapid microwave-assisted henry reaction in solvent-free processes. Synlett.

[14] Tanaka K., Hachiken S. (2008). Enantioselective Henry reaction catalyzed by trianglamine–Cu (OAc)_2_ complex under solvent-free conditions. Tetrahedron Lett..

[15] Reddy K.R., Rajasekhar C.V., Krishna G.G. (2007). Zinc-proline complex: An efficient, reusable catalyst for direct nitroaldol reaction in aqueous media. Synth. Commun..

[16] Ren Y., Cai C. (2007). Iodine catalysis in aqueous medium: An improved reaction system for Knoevenagel and Nitroaldol condensation. Catalysis Lett..

[17] Busto E., Gotor-Fernández V., Gotor V. (2010). Protein-mediated nitroaldol addition in aqueous media. Catalytic promiscuity or unspecific catalysis?. Org. Process Res. Dev..

[18] Purkarthofer T., Gruber K., Gruber-Khadjawi M., Waich K., Skranc W., Mink D., Griengl H. (2006). A biocatalytic Henry reaction—The hydroxynitrile lyase from Hevea brasiliensis also catalyzes nitroaldol reactions. Angew. Chem. Int. Ed..

[19] Tang R.-C., Guan Z., He Y.-H., Zhu W. (2010). Enzyme-catalyzed Henry (nitroaldol) reaction. J. Mol. Catal. B: Enzym..

[20] Gruber-Khadjawi M., Purkarthofer T., Skranc W., Griengl H. (2007). Hydroxynitrile lyase-catalyzed enzymatic nitroaldol (Henry) reaction. Adv. Synth. Catal..

[21] Humble M.S., Berglund P. (2011). Biocatalytic promiscuity. Eur. J. Org. Chem..

[22] Kapoor M., Gupta M.N. (2012). Lipase promiscuity and its biochemical applications. Process Biochem..

[23] Le Z.-G., Guo L.-T., Jiang G.-F., Yang X.-B., Liu H.-Q. (2013). Henry reaction catalyzed by Lipase A from Aspergillus niger. Green Chem. Lett. Rev..

[24] Xue L., Qiu H., Li Y., Lu L., Huang X., Qu Y. (2011). A novel water-in-ionic liquid microemulsion and its interfacial effect on the activity of laccase. Colloids Surf. B.

[25] Moniruzzaman M., Kamiya N., Goto M. (2008). Biocatalysis in water-in-ionic liquid microemulsions: A case study with horseradish peroxidase. Langmuir.

[26] Pavlidis I.V., Gournis D., Papadopoulos G.K., Stamatis H. (2009). Lipases in water-in-ionic liquid microemulsions: Structural and activity studies. J. Mol. Catal. B: Enzym..

[27] Xue L., Li Y., Zou F., Lu L., Zhao Y., Huang X., Qu Y. (2012). The catalytic efficiency of lipase in a novel water-in-[Bmim][PF_6_] microemulsion stabilized by both AOT and Triton X-100. Colloids Surf. B.

[28] Gao Y., Han S., Han B., Li G., Shen D., Li Z., Du J., Hou W., Zhang G. (2005). TX-100/water/1-butyl-3-methylimidazolium hexafluorophosphate microemulsions. Langmuir.

